# Psychosocial work exposures and suicide ideation: a study of multiple exposures using the French national working conditions survey

**DOI:** 10.1186/s12889-020-09019-3

**Published:** 2020-06-09

**Authors:** Isabelle Niedhammer, Maryline Bèque, Jean-François Chastang, Sandrine Bertrais

**Affiliations:** 1 INSERM, Univ Angers, Univ Rennes, EHESP, Irset (Institut de recherche en santé, environnement et travail) - UMR_S 1085, ESTER Team, 28 rue Roger Amsler, CS 74521, 49045, Angers, France; 2 DARES, Ministère du Travail, Paris, France

**Keywords:** Suicide ideation, Psychosocial work factors, Job stress, Working conditions, Occupational exposures

## Abstract

**Background:**

Our study aimed to explore the associations between psychosocial work exposures, as well as other occupational exposures, and suicide ideation in the French national working population. An additional objective was to study the cumulative role of occupational exposures in this outcome.

**Methods:**

The study was based on a nationally representative sample of the French working population of 20,430 employees, 8579 men and 11,851 women (2016 French national Working Conditions survey). Occupational exposures included 21 psychosocial work factors, 4 factors related to working time/hours and 4 factors related to the physical work environment. Suicide ideation within the last 12 months was the outcome. The associations between exposures and outcome were studied using weighted logistic regression models adjusted for covariates.

**Results:**

The 12-month prevalence of suicide ideation was 5.2% among men and 5.7% among women. Among the occupational exposures, psychosocial work factors were found to be associated with suicide ideation: quantitative and cognitive demands, low influence and possibilities for development, low meaning at work, low sense of community, role conflict, job insecurity, temporary employment, changes at work, and internal violence. Some rare differences in these associations were observed between genders. Linear associations were observed between the number of psychosocial work exposures and suicide ideation.

**Conclusions:**

Psychosocial work factors were found to play a major role in suicide ideation, and their effects were cumulative on this outcome. More research on multiple and cumulative exposures and suicide ideation and more prevention towards the psychosocial work environment are needed.

## Background

Suicide has become a major issue in occupational health, as although the rate of suicide is higher among unemployed and economically inactive people than among employed people, the total number of suicides is higher in the working population than in the non-working population [1]. Furthermore, a substantial body of the literature was published on the associations between occupation and suicide, and showed that some occupations were at higher risk of suicide than others [2, 3]. Nevertheless, the literature appears scarce on the factors that may explain the differences in suicide risk between occupations.

Psychosocial work factors may have detrimental effects on health, particularly on mental health. Literature reviews and meta-analyses underlined the associations between psychosocial work factors and various mental health outcomes, especially those related to depression [4–8]. It is thus legitimate to question whether psychosocial work factors may have effects on suicide. To date, two systematic literature reviews were performed, the first one on the association between workplace bullying and suicide ideation [9], and the second one on the associations between the other psychosocial work factors and suicidality [10].

The first review [9] by Leach et al., published in 2017, showed an association between workplace bullying and suicide ideation that was found in 8 studies among the 12 included studies. The second review [10], more comprehensive, that used a meta-analysis, by Milner et al., published in 2018, found that the studied psychosocial work factors were associated with suicide ideation among the 14 included studies. The factors from the job strain model, the most recognized and used theoretical model of job stress, were significantly associated with suicide ideation: high job demands, low job control, job strain (the combination of high demands and low control), and low social support. Two other factors, less studied, were also observed as risk factors: effort-reward imbalance and job insecurity. There was also one included study that reported a significant association between role conflict and suicide ideation. Nevertheless, the authors of these two reviews [9, 10] concluded to a lack of high-quality studies and the need for more studies in this area.

Our study is thus a contribution to the literature on the topic of psychosocial work factors in association with suicide ideation. Contrarily to previous studies, it had the advantage to overcome some previous limitations: indeed, we used a large nationally representative sample of the working population and not a sample related to a specific occupation/work sector, we studied both men and women, we explored a large range of psychosocial work factors and not a limited number of factors, and, above all, we examined multiple exposure to these factors, which has never been done to date.

The objectives of this study were to explore the associations of psychosocial work factors, as well as other occupational exposures, with suicide ideation, and also to assess the effects of multiple exposure to these factors.

## Methods

The study relied on the data of the last version of the French national Working Conditions survey performed by DARES (Direction de l’Animation de la Recherche, des Études et des Statistiques) of the French Ministry of Labour in 2016. It was based on a nationally representative sample of the working population aged 15 or more. This sample was selected randomly using a two-stage sampling design, with two successive selections of households and workers (if more than one worker in the household). Data were collected using both a face-to-face interview and a self-administered questionnaire, that was used to collect items on more sensitive issues (suicide ideation, social support outside work, life events, and psychosocial work factors).

Suicide ideation was the outcome and was measured using one item related to suicide thoughts within the last 12 months. The item was the following: ‘Within the last 12 months, have you thought about suicide?’, that was used in another French national survey, in the general population, the Health Barometer [11].

Occupational factors included 4 groups of factors:

- 21 psychosocial work factors, that were assessed using 79 items, inspired from various concepts including those of the COPSOQ questionnaire (Copenhagen Psychosocial Questionnaire), and grouped into 5 topics:

1. Demands at work: quantitative demands (7 items), cognitive demands (3 items), emotional demands (2 items), demands for hiding emotions (2 items).
2. Work organization and job content: influence at work (6 items), degree of freedom (3 items), possibilities for development (5 items), meaning of work (3 items).
3. Interpersonal relations and leadership: social support from supervisors and colleagues (7 items), sense of community (3 items), quality of leadership (5 items), predictability (2 items), role clarity (2 items), role conflict (9 items).
4. Work-individual interface: job satisfaction (3 items), work-family conflict (2 items), job insecurity (2 items), changes at work (2 items), temporary employment (1 item).
5. Workplace violence: internal violence at work (i.e. from colleagues, supervisors, etc.) (6 items), external violence at work (i.e. from the public, patients, clients, customers, etc.) (4 items).

The score for each factor was calculated from the sum of the items and dichotomized at the median of the distribution in the total sample, and these binary variables were summed up to construct multiple exposure for each topic and for all psychosocial work factors together.

- 4 working time/hours factors: long working hours (1 item: working more than 48 h per week, following the European directive on working time), night work (1 item: working between midnight and 5 am at least 50 nights a year), shift work (1 item, working on alternating/rotating shifts), and unsocial work days (1 item, working on Saturday or Sunday at least 40 times a year).
- 4 physical work factors: biomechanical exposure (7 items: long-term exposure to standing, difficult or tiring position, walking, heavy loads, painful or tiring movements, vibrations, repetitive tasks), exposure to fumes or dust (1 item), exposure to toxic and dangerous products (1 item), and exposure to noise (1 item).

Multiple exposure to working time/hours factors and to physical work factors were also constructed.

The following covariates were taken into account: age, marital status (1 item: living with or without a partner), social support outside work (2 items: having someone to rely on to discuss personal issues or take a difficult decision, and need more help than help received), life events before the age of 18 and within the last 3 years (among the following items: serious health problems of oneself or close family member, death of close family member, family conflict, and exposure to violence), occupation and economic activity of the company.

The differences between men and women were explored, and following Doyal’s definition of gender [12], the term ‘gender’ was used instead of ‘sex’ as these differences may be related to biological but also social aspects. Consequently, all analyses were done for men and women separately.

The statistical analyses were performed using weighted data in order to take non-response and marginal calibration into account. Firstly, comparison was done between genders for all studied variables using the Rao-Scott Chi-2 test. The prevalence of suicide ideation was also compared between groups using the same test. Secondly, weighted logistic regression models were used to study the associations between occupational factors and suicide ideation. Three types of model were performed: (1) the first one explored the crude association between each occupational factor and suicide ideation (unadjusted models), (2) the second one additionally adjusted for the forementioned covariates (adjusted models), and (3) the third one explored the association between multiple exposure and suicide ideation with adjustment for covariates. Trend tests were performed for this last model. Sensitivity analyses explored the robustness of the results: (1) with additional adjustment for full/part time work, public/private sector, company size, and chronic disease, and (2) with additional adjustment for working time/hours and physical work factors in the study of multiple exposure to psychosocial work factors. Statistical gender-related interactions were also tested among the total sample in the models adjusted for covariates to explore potential differences in the exposure-outcome associations between genders and determine their statistical significance.

We used SAS software to perform all statistical analyses.

## Results

The rates of participation to the face-to-face interview (74%) and of response to the self-administered questionnaire (94%) were both high. Among the 27,610 participants, 20,430 people were employees, aged 15–65, working at the time of the survey, and responded to the self-administered questionnaire. Thus, the study sample included 20,430 employees, 8579 men and 11,851 women. The description of the sample is presented in Table 1 for the covariates. Significant statistical differences between genders were observed for all covariates except age. Women were more likely to live alone, to have low social support outside work, to report life events, and to work as clerks/service workers and associate professionals/technicians, and to work in the services than men. Significant statistical differences in the prevalence of exposure to occupational factors were also found between genders (not showed). Women were more likely to be exposed to most psychosocial work factors (emotional demands, demands for hiding emotions, low influence, low degree of freedom, low possibilities for development, low job satisfaction, external violence, and job insecurity) than men. Women were also more likely to be exposed to unsocial work days than men. In contrast, men were more likely to be exposed to low meaning of work, role conflict, low predictability, as well as to long working hours, night work, and shift work, and to all physical work exposures than women.

**Table 1:** Description of the study sample and prevalence of suicide ideation according to covariates

	Men				Women			
			Suicide ideation ^a^				Suicide ideation ^a^	
	n	% ^b^	% ^b^	*p*-value ^c^	n	% ^b^	% ^b^	*p*-value ^c^
All	8579	–	5.2		11,851	–	5.7	
Age (years)				0.128				0.360
< 30	1020	19.0	4.4		1283	18.1	4.7	
30–39	1864	25.5	3.7		2473	23.6	4.7	
40–49	2799	28.6	6.9		3820	28.1	6.0	
≥ 50	2896	26.9	5.4		4275	30.2	6.8	
Marital status				0.002				0.015
Living with a partner	6827	76.2	4.3		8849	72.6	5.0	
Alone	1752	23.8	8.0		3001	27.4	7.7	
Social support outside work				< 0.001				< 0.001
Yes and don’t need more help	6924	81.7	3.7		8939	78.5	3.0	
No and don’t need more help	340	4.2	8.5		353	2.9	9.7	
Yes but need more help	1006	12.0	12.0		1952	15.3	15.0	
No and need more help	191	2.0	16.4		432	3.3	23.7	
Life event(s) during childhood				< 0.001				< 0.001
None	4154	48.1	2.5		4608	38.5	2.9	
1	2462	28.4	4.7		3556	30.1	3.2	
≥ 2	1926	23.6	11.3		3644	31.3	11.6	
Life event(s) within the last 3 years				< 0.001				< 0.001
None	4675	56.4	3.0		4854	42.6	2.1	
1	2588	29.4	5.3		3945	32.1	5.3	
≥ 2	1286	14.2	13.6		3012	25.3	12.3	
Occupation				0.686				0.725
Managers, professionals	2025	22.4	5.1		1691	14.9	4.9	
Associate professionals, technicians	2402	25.9	4.4		3978	27.9	5.2	
Clerks, service workers	1397	14.4	6.1		5357	48.3	6.1	
Blue-collar workers	2681	37.3	5.5		751	8.9	6.6	
Economic activity				0.271				0.788
Services	5993	66.2	5.6		10,866	90.1	5.7	
Others	2579	33.8	4.5		979	9.9	6.1	

^a^Data on suicide ideation within the last 12 months were available for 8543 men and 11,795 women

^b^Weighted percentages

^c^*p-*value for the comparison of the weighted prevalence of suicide ideation according to covariates (Rao-Scott Chi-square test)

The prevalence of suicide ideation was 5.2% for men and 5.7% for women, without any statistical difference between genders. The prevalence of suicide ideation according to covariates is presented in Table 1. This prevalence increased among people living alone, with low social support outside work and life events. There was no statistical difference between age groups, occupations and economic activities of the company.

Table 2 presents the associations between each occupational factor and suicide ideation among men. The associations were significant for all psychosocial work factors before adjustment for covariates except for cognitive demands, degree of freedom, predictability, and temporary employment. After adjustment for covariates, the number of significant associations was lower, but the associations remained significant for quantitative demands, low possibilities for development, low meaning, low sense of community, low job satisfaction, job insecurity, temporary employment, and internal violence.

**Table 2:** Associations of occupational exposures with suicide ideation in men. Results from weighted logistic regression analyses (each occupational factor studied separately)

Men (*N* = 7781) ^a^	Unadjusted				Adjusted for covariates ^c^			
	OR	95% CI		*p*-value	OR	95% CI		*p*-value
**Psychosocial work factors**^b^								
*Demands at work*								
High quantitative demands	**2.00**	**1.32**	**3.03**	**0.001**	**1.74**	**1.10**	**2.76**	**0.018**
High cognitive demands	0.90	0.61	1.33	0.595	0.79	0.52	1.22	0.288
High emotional demands	**1.59**	**1.06**	**2.38**	**0.024**	1.29	0.81	2.07	0.283
High demands for hiding emotions	**2.51**	**1.66**	**3.79**	**< 0.001**	1.59	0.97	2.58	0.064
*Work organization and job content*								
Low influence	**1.54**	**1.03**	**2.30**	**0.036**	1.43	0.94	2.17	0.091
Low degree of freedom	1.33	0.89	2.00	0.168	1.05	0.69	1.59	0.827
Low possibilities for development	**2.41**	**1.58**	**3.67**	**< 0.001**	**1.98**	**1.20**	**3.26**	**0.007**
Low meaning of work	**2.29**	**1.46**	**3.59**	**< 0.001**	**1.85**	**1.13**	**3.03**	**0.015**
*Interpersonal relations and leadership*								
Low predictability	0.88	0.57	1.34	0.542	0.85	0.54	1.34	0.486
Low role clarity	**1.62**	**1.08**	**2.42**	**0.019**	1.34	0.86	2.07	0.194
High role conflict	**1.84**	**1.16**	**2.91**	**0.009**	1.43	0.90	2.26	0.133
Low quality of leadership	**2.00**	**1.29**	**3.09**	**0.002**	1.56	0.99	2.46	0.055
Low social support	**1.60**	**1.01**	**2.55**	**0.045**	1.30	0.79	2.12	0.301
Low sense of community	**2.35**	**1.49**	**3.70**	**< 0.001**	**1.91**	**1.19**	**3.06**	**0.007**
*Work-individual interface*								
Low job satisfaction	**2.18**	**1.40**	**3.40**	**0.001**	**1.71**	**1.06**	**2.76**	**0.027**
Work-family conflict	**1.62**	**1.08**	**2.43**	**0.021**	1.44	0.90	2.31	0.130
High job insecurity	**2.78**	**1.88**	**4.12**	**< 0.001**	**2.25**	**1.44**	**3.50**	**< 0.001**
High changes at work	**1.89**	**1.22**	**2.93**	**0.005**	1.37	0.87	2.16	0.177
Temporary employment	1.79	0.90	3.56	0.095	**2.18**	**1.04**	**4.56**	**0.038**
*Workplace violence*								
High internal violence	**2.48**	**1.57**	**3.92**	**< 0.001**	**1.69**	**1.08**	**2.65**	**0.022**
High external violence	**1.56**	**1.04**	**2.34**	**0.033**	1.20	0.74	1.94	0.458
**Working time/hours factors**								
Long working hours (> 48 h/week)	0.74	0.44	1.24	0.249	0.76	0.42	1.38	0.371
Night work (> 50/year)	1.00	0.53	1.89	0.998	0.83	0.40	1.69	0.599
Unsocial work days (> 40/year)	0.88	0.54	1.43	0.594	0.61	0.37	1.02	0.061
Shift work	1.19	0.64	2.24	0.584	1.28	0.62	2.66	0.504
**Physical work exposures**								
High biomechanical exposure ^b^	0.96	0.64	1.42	0.821	0.63	0.38	1.04	0.073
Fumes and dust exposure	0.97	0.65	1.45	0.871	0.84	0.53	1.34	0.466
Toxic and dangerous products exposure	0.93	0.62	1.38	0.707	0.81	0.55	1.22	0.317
Noise exposure	1.22	0.79	1.88	0.381	1.08	0.69	1.69	0.731

Odds-Ratio (OR) and 95% confidence interval (CI)

^a^Reported results are those from complete case analyses that included participants with no missing data for the variables of interest (suicide ideation, all occupational exposures, covariates). The observed associations were similar using all available data for each occupational exposure

^b^Median cut-off of the total sample was used to classify workers in low or high exposure groups

^c^Adjusted for age, marital status, social support outside work, life events before the age of 18, life events within the last 3 years, occupation, and economic activity of the company

Table 3 presents the associations between each occupational factor and suicide ideation among women. Before adjustment for covariates, almost all psychosocial work factors were significantly associated with suicide ideation, except possibilities for development, predictability, work-family conflict, and temporary employment. After adjustment for covariates, the number of significant associations was reduced, but we observed that exposure to quantitative demands, cognitive demands, low influence, low meaning, role conflict, changes at work, and internal violence were risk factors of suicide ideation.

**Table 3:** Associations of occupational exposures with suicide ideation in women. Results from weighted logistic regression analyses (each occupational factor studied separately)

Women (*N* = 10,666) ^a^	Unadjusted				Adjusted for covariates ^c^			
	OR	95% CI		*p*-value	OR	95% CI		*p*-value
**Psychosocial work factors**^b^								
*Demands at work*								
High quantitative demands	**1.88**	**1.31**	**2.69**	**0.001**	**1.50**	**1.02**	**2.20**	**0.038**
High cognitive demands	**1.97**	**1.35**	**2.86**	**< 0.001**	**1.74**	**1.18**	**2.59**	**0.006**
High emotional demands	**1.71**	**1.11**	**2.63**	**0.014**	1.41	0.87	2.28	0.165
High demands for hiding emotions	**2.33**	**1.45**	**3.72**	**< 0.001**	1.44	0.89	2.34	0.134
*Work organization and job content*								
Low influence	**1.49**	**1.06**	**2.10**	**0.023**	**1.47**	**1.03**	**2.09**	**0.034**
Low degree of freedom	**1.54**	**1.06**	**2.23**	**0.025**	1.36	0.93	1.97	0.109
Low possibilities for development	1.39	0.96	2.00	0.080	1.12	0.77	1.62	0.548
Low meaning of work	**2.04**	**1.45**	**2.88**	**< 0.001**	**1.66**	**1.15**	**2.40**	**0.007**
*Interpersonal relations and leadership*								
Low predictability	1.08	0.77	1.53	0.648	1.08	0.73	1.58	0.708
Low role clarity	**1.80**	**1.27**	**2.54**	**0.001**	1.37	0.96	1.97	0.086
High role conflict	**2.54**	**1.77**	**3.63**	**< 0.001**	**1.79**	**1.23**	**2.61**	**0.002**
Low quality of leadership	**1.87**	**1.30**	**2.71**	**0.001**	1.37	0.94	1.98	0.102
Low social support	**2.05**	**1.37**	**3.06**	**< 0.001**	1.42	0.95	2.14	0.089
Low sense of community	**2.07**	**1.39**	**3.09**	**< 0.001**	1.44	0.97	2.14	0.067
*Work-individual interface*								
Low job satisfaction	**1.89**	**1.32**	**2.70**	**< 0.001**	1.26	0.85	1.87	0.242
Work-family conflict	1.31	0.92	1.86	0.132	1.08	0.76	1.54	0.666
High job insecurity	**1.61**	**1.14**	**2.28**	**0.007**	1.17	0.80	1.71	0.419
High changes at work	**2.37**	**1.64**	**3.43**	**< 0.001**	**1.50**	**1.01**	**2.25**	**0.046**
Temporary employment	1.06	0.59	1.92	0.839	1.02	0.53	1.97	0.953
*Workplace violence*								
High internal violence	**3.02**	**1.99**	**4.59**	**< 0.001**	**2.11**	**1.38**	**3.23**	**0.001**
High external violence	**1.64**	**1.16**	**2.32**	**0.005**	1.42	0.99	2.02	0.056
**Working time/hours factors**								
Long working hours (> 48 h/week)	1.56	0.84	2.90	0.163	1.49	0.78	2.88	0.230
Night work (> 50/year)	**0.29**	**0.13**	**0.65**	**0.003**	**0.29**	**0.13**	**0.66**	**0.003**
Unsocial work days (> 40/year)	0.86	0.57	1.29	0.472	0.93	0.61	1.44	0.760
Shift work	0.99	0.31	3.09	0.980	0.87	0.22	3.41	0.837
**Physical work exposures**								
High biomechanical exposure ^b^	1.39	0.98	1.97	0.062	1.15	0.77	1.72	0.498
Fumes and dust exposure	1.29	0.90	1.85	0.169	1.02	0.66	1.55	0.940
Toxic and dangerous products exposure	1.02	0.72	1.46	0.898	0.87	0.58	1.29	0.479
Noise exposure	**1.98**	**1.26**	**3.11**	**0.003**	1.68	0.99	2.82	0.053

Odds-Ratio (OR) and 95% confidence interval (CI)

^a^Reported results are those from complete case analyses that included participants with no missing data for the variables of interest (suicide ideation, all occupational exposures, covariates). The observed associations were similar using all available data for each occupational exposure

^b^Median cut-off of the total sample was used to classify workers in low or high exposure groups

^c^Adjusted for age, marital status, social support outside work, life events before the age of 18, life events within the last 3 years, occupation, and economic activity of the company

In the analyses of the total sample of men and women, two significant gender-related interaction terms were found and showed that the association between cognitive demands and suicide ideation was significant among women and not among men (*p* = 0.001), and that the association between job insecurity and suicide ideation was significant among men and not among women (*p* = 0.040).

For both men and women, no exposure related to working time/hours and the physical work environment was associated with suicide ideation (Tables 2-3). Among women, night work was found to be a protective factor for suicide ideation, and noise exposure that was associated with suicide ideation before adjustment for covariates, was borderline significant after this adjustment.

**Fig. 1:**
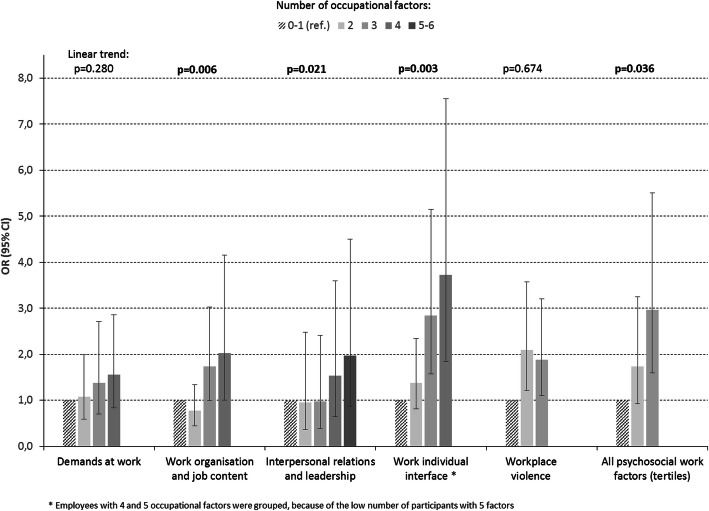
Multiple occupational exposures and suicide ideation in men: odds-ratio (OR) and 95% confidence interval (CI) after adjustment for covariates

**Fig. 2:**
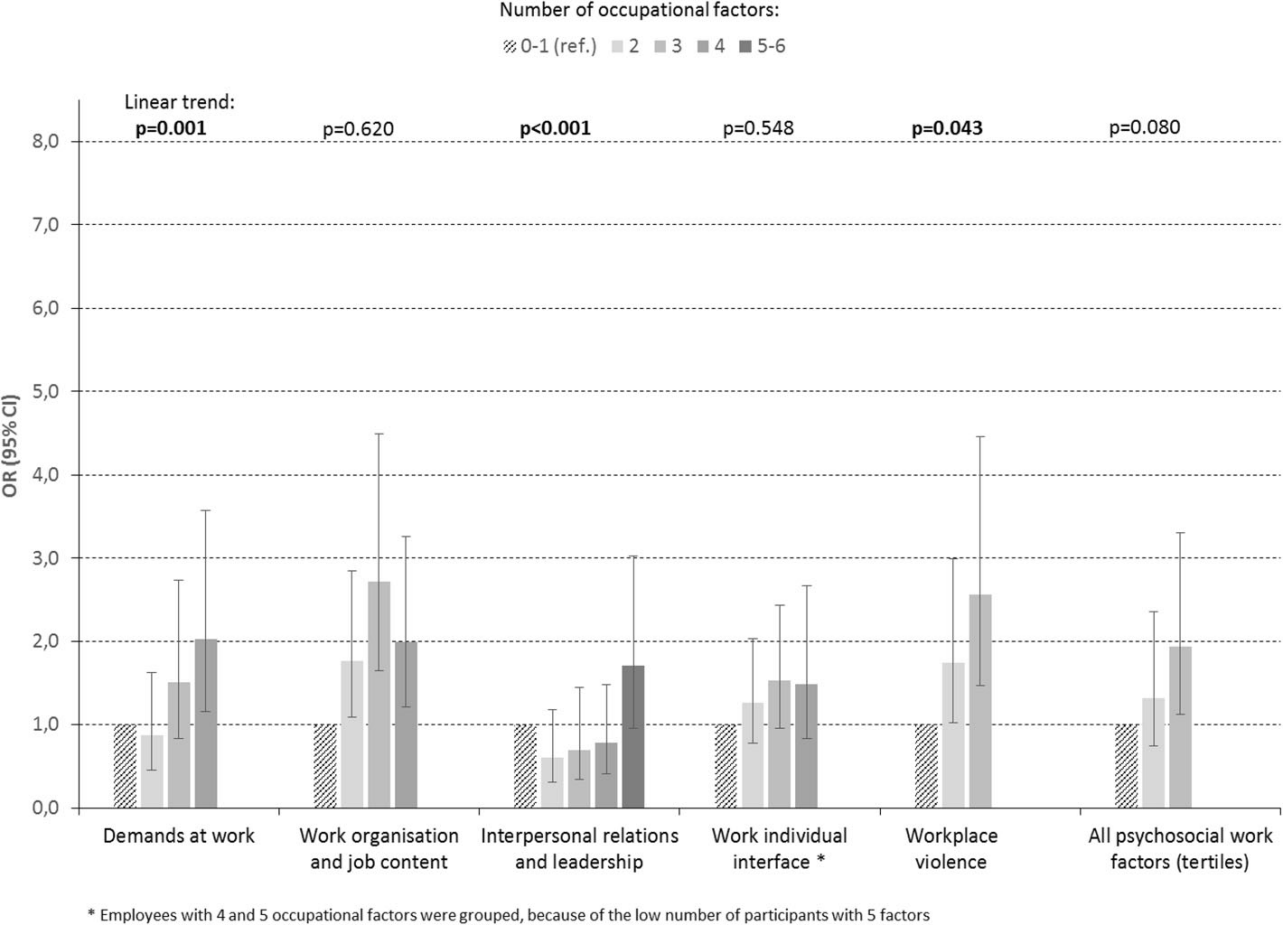
Multiple occupational exposures and suicide ideation in women: odds-ratio (OR) and 95% confidence interval (CI) after adjustment for covariates

Figures 1-2 show the results for multiple exposure in association with suicide ideation. Significant trend tests were observed for work organization and job content, interpersonal relations and leadership, and work-individual interface among men and for demands at work, interpersonal relations and leadership, and workplace violence among women. Trend tests for all psychosocial work factors together were significant for men and borderline significant among women. No interaction was found between gender and the number of psychosocial work factors in association with suicide ideation, suggesting no difference in linear trend between genders. No trend test was found to be significant for multiple exposure to working time/hours and physical work exposures (not showed).

## Discussion

### Summary of the results

The study is one of the first to provide a comprehensive view of the associations between occupational factors and suicide ideation. It showed that psychosocial work factors were associated with suicide ideation and that multiple exposure to these factors increased the risk of suicide ideation linearly. No factor related to working time/hours and the physical work environment was found to increase the risk of suicide ideation.

### Comparison with the literature

The number of the previous studies on the associations between psychosocial work factors and suicide ideation is low. The literature reviews published recently showed that there were 14 studies that explored psychosocial work factors and suicide ideation [10] and 8 studies specifically on workplace bullying and suicide ideation [9]. Our results are in agreement with these reviews for job demands (quantitative and cognitive demands in our study), low job control (low influence, low possibilities for development), low social support (low sense of community), job insecurity, role conflict, and workplace bullying (internal violence). Our findings related to temporary employment are also in line with the results of two studies that showed that employment arrangement, in particular casual/fixed term employment, was associated with thoughts about suicide [13] and that precarious work was associated with suicidal ideation [14]. We found that low meaning at work and changes at work increased the risk of suicide ideation, results that have never been observed before. Our study showed that there was a linear association between multiple exposure to psychosocial work factors and suicide ideation, and our study may be the first one to demonstrate the cumulative effects of these factors on suicide ideation. We found no association between exposures related to working time/hours and the physical work environment and suicide ideation. Other authors did not find any association between working time/hours and suicide ideation [15, 16]. To our knowledge, information is missing on the associations between occupational exposures of physical nature and suicide ideation.

### Strengths and limitations of the study

The study had many strengths. It was based on a large nationally representative sample of the working population with high participation and response rates. It used weights in order to provide results that could be extrapolated to the whole population. Men and women were studied separately and differences between genders including gender-related interactions were tested following the best practices [17, 18]. We found that there were significant differences between genders in covariates and occupational exposures, in most cases, at the expense of women. Nevertheless, the prevalence of suicide ideation was not statistically different between men and women, and most of the exposure-outcome associations were similar between genders. There were, however, two exceptions for cognitive demands, observed as a risk factor among women only, and job insecurity, found as a risk factor among men only. This last association might be explained by the role of sole or main bread winner in the family taken by men. Suicide ideation was measured using one item already used in another national French survey (the Health Barometer), that found a similar 12-month prevalence of suicide ideation (3.9% in 2009–2010 in France) [11]. The study included a large number of occupational factors, especially psychosocial work factors. We also studied other occupational exposures related to working time/hours and physical exposures, that were not associated with suicide ideation. As our assumption was that psychosocial work factors would be more strongly associated with the outcome than the other occupational exposures, our results are thus consistent with what was expected. One of the major assets was the study of multiple exposures that has never been done before in association with suicide ideation. Important covariates were taken into account in the analyses and sensitivity analyses were performed that confirmed the robustness of the results.

The study had, however, some limitations. It was a cross-sectional study, consequently no causal conclusion could be drawn from the results and reverse causation was possible. A healthy worker effect might have selected the healthiest people at the workplace and at the most exposed jobs and led to an underestimation of the observed associations. This effect may be a potential explanation of the protective association between night work and suicide ideation found in our study. No validated questionnaire was used for the measurement of suicide ideation, and the item used did not include the frequency of thoughts. This single item may also have led to a recall bias. No validated questionnaire was used for the measurement of occupational factors, and some occupational factors may be missing such as organizational injustice. As the study relied on self-reported data, a reporting bias might be suspected that could lead to an overestimation of the associations. Some covariates may be missing such as history of mental disorders. Nevertheless, adjusting for mental disorders may lead to overadjustment, as these disorders, especially depression, are likely to be in the causal pathway between occupational exposures and suicide ideation. Indeed, depression is a major risk factor of suicidal behaviour and may thus explain the observed associations between psychosocial work factors and suicide ideation in our study.

## Conclusions

Our study suggested that psychosocial work exposures may play a major role in suicide ideation, and the role of the other occupational exposures might be negligible in this outcome. Our results also showed that various psychosocial work factors were risk factors for suicide ideation, in particular those related to job demands, job control, interpersonal relationships including workplace violence, job insecurity, temporary employment, and changes at work. There were some rare differences in these risk factors between men and women. Finally, our study highlighted the cumulative role of psychosocial work factors in suicide ideation. More research is needed to confirm our results, and more prevention towards the psychosocial work environment may be useful to prevent suicide ideation among the working population. Our study may help and guide clinical practice to better identify the factors at the workplace that may increase the risk of suicide ideation. More attention should also be paid to the accumulation of these factors that may increase the risk still further.

## Data Availability

The dataset used and analysed during the current study are available from the corresponding author on reasonable request.
